# Predicting Success of a Digital Self-Help Intervention for Alcohol and Substance Use With Machine Learning

**DOI:** 10.3389/fpsyg.2021.734633

**Published:** 2021-09-03

**Authors:** Lucas A. Ramos, Matthijs Blankers, Guido van Wingen, Tamara de Bruijn, Steffen C. Pauws, Anneke E. Goudriaan

**Affiliations:** ^1^Department of Psychiatry, Amsterdam UMC, and Amsterdam Institute for Addiction Research, University of Amsterdam, Amsterdam, Netherlands; ^2^Arkin Mental Health Care, Amsterdam, Netherlands; ^3^Trimbos Institute, The Netherlands Institute of Mental Health and Addiction, Utrecht, Netherlands; ^4^Jellinek Prevention, Amsterdam, Netherlands; ^5^Department of Communication and Cognition, Tilburg University, Tilburg, Netherlands; ^6^Department of Remote Patient Management and Chronic Care, Philips Research, Eindhoven, Netherlands; ^7^Amsterdam Public Health Research Institute, Amsterdam, Netherlands

**Keywords:** machine learning, eHealth, ATOD, Substance Use Disorder, addiction, log data analysis, CBT

## Abstract

**Background:**

Digital self-help interventions for reducing the use of alcohol tobacco and other drugs (ATOD) have generally shown positive but small effects in controlling substance use and improving the quality of life of participants. Nonetheless, low adherence rates remain a major drawback of these digital interventions, with mixed results in (prolonged) participation and outcome. To prevent non-adherence, we developed models to predict success in the early stages of an ATOD digital self-help intervention and explore the predictors associated with participant’s goal achievement.

**Methods:**

We included previous and current participants from a widely used, evidence-based ATOD intervention from the Netherlands (Jellinek Digital Self-help). Participants were considered successful if they completed all intervention modules and reached their substance use goals (i.e., stop/reduce). Early dropout was defined as finishing only the first module. During model development, participants were split per substance (alcohol, tobacco, cannabis) and features were computed based on the log data of the first 3 days of intervention participation. Machine learning models were trained, validated and tested using a nested k-fold cross-validation strategy.

**Results:**

From the 32,398 participants enrolled in the study, 80% of participants did not complete the first module of the intervention and were excluded from further analysis. From the remaining participants, the percentage of success for each substance was 30% for alcohol, 22% for cannabis and 24% for tobacco. The area under the Receiver Operating Characteristic curve was the highest for the Random Forest model trained on data from the alcohol and tobacco programs (0.71 95%CI 0.69–0.73) and (0.71 95%CI 0.67–0.76), respectively, followed by cannabis (0.67 95%CI 0.59–0.75). Quitting substance use instead of moderation as an intervention goal, initial daily consumption, no substance use on the weekends as a target goal and intervention engagement were strong predictors of success.

**Discussion:**

Using log data from the first 3 days of intervention use, machine learning models showed positive results in identifying successful participants. Our results suggest the models were especially able to identify participants at risk of early dropout. Multiple variables were found to have high predictive value, which can be used to further improve the intervention.

## Introduction

Alcohol, tobacco, and other drugs (ATOD) use are among the leading risk factors for morbidity and mortality worldwide (Degenhardt et al., 2013; Shield et al., 2016; Volkow and Boyle, 2018) and can be a major cause of negative social, economic, and medical effects (Degenhardt and Hall, 2012). Digital self-help interventions for ATOD use have been broadly explored as a tool to help mitigate substance use and related harm, often with positive results (Riper et al., 2008; Tait et al., 2014; Mujcic et al., 2018; Berman et al., 2019; Olthof et al., 2021). Nonetheless, participant adherence remains a major issue in digital interventions for mental disorders, either due to not using the intervention (non-adherence) or due to not completing follow-up measures (study dropout) (Khadjesari et al., 2014). Outside randomized controlled trials, dropout rates reported in the literature for digital health interventions vary from 44% in a digital intervention for amphetamine-type stimulant abuse, up to 83% for an internet-based intervention for psychological disorders (Melville et al., 2010; Tait et al., 2014).

Different types of data can be used to predict adherence or dropout in eHealth interventions. A study by Symons et al. (2019) focused on the prediction of outcome in cognitive behavioral therapy, and used variables related to demographics, medical history, psychiatric history, and symptoms of alcohol dependence. Using patient demographics and log data variables, the study of Pedersen et al. (2019) focused on the prediction of dropouts from an intervention for chronic lifestyle diseases (such as diabetes, heart disease, chronic obstructive pulmonary disease, and cancer), and reported an AUC of 0.92 with log data variables being more predictive than demographics.

Log data often shows high predictive value. It consists of records of actions performed by the user when using the intervention and can provide new insights into the actual usage of each individual module (Sieverink et al., 2017a). Log data was used for the prediction of outcome (dietary changes) of an online intervention for eating disorders (Manwaring et al., 2008), the results suggested certain variables (e.g., the number of weeks the intervention was used, accessing content pages, and posting in the journals) were significantly associated with dietary changes.

Cognitive Behavioral Therapy (CBT) and motivational interviewing (MI) based digital self-help interventions have been developed to help treat people suffering from diverse conditions including problem drinking (Riper et al., 2008; Mujcic et al., 2020), tobacco smoking (Mujcic et al., 2018), and cannabis (Olthof et al., 2021). Some central elements from CBT self-help interventions are: exploring and exploiting ambiguity regarding behavior change, stimulus control, stress management, social support, goal setting and pursuit through monitoring and exercises (Foreyt and Poston, 1998). Retaining users and increasing engagement has always been a priority of self-help health interventions (Freyne et al., 2012) since participant adherence plays a major role in the success of an intervention (Sieverink et al., 2017b).

The large number of variables available in log data, that are collected during a digital self-help intervention make machine learning especially suitable for the prediction task since they are capable of handling high-dimensional data and discover previously unknown relationships between variables by adding non-linearity to the learning process.

In this study, we aim to use machine learning models to predict participant success using data from the early stages of a digital self-help intervention for problematic substance use (alcohol, tobacco, or cannabis) and explore the components that are associated with the success of participants in reaching the goals that they set at the start of the intervention. We also explored model interpretability, since understanding the factors associated with participant adherence can subsequently lead to implementation research, investigating changes to improve relevant adherence patterns, and thus, improve success rates of the intervention (Acion et al., 2017).

## Materials and Methods

### Jellinek Intervention

**FIGURE 1:**
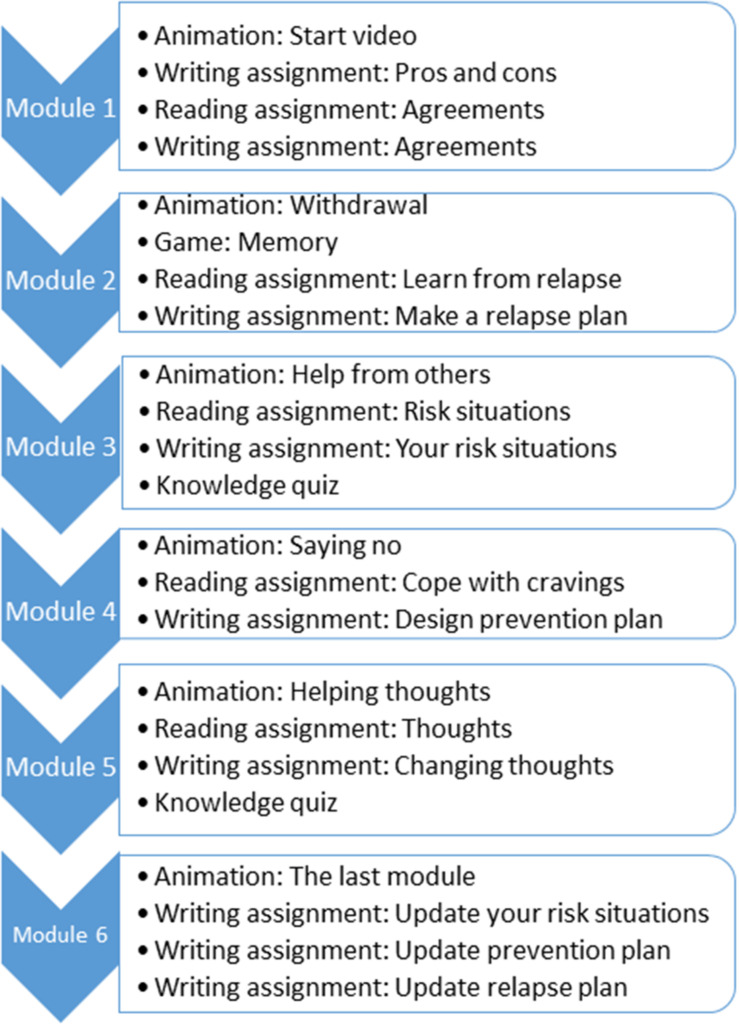
Diagram with the activities of each module of the Jellinek intervention. Each module should last at least 5 days. During each module the participants are encouraged to register their daily consumption.

We included all participants enrolled between January 2016 and October 2020 in a widely used, evidence-based unguided digital self-help intervention for alcohol, cannabis, and cocaine use, tobacco smoking, and gambling (Jellinek Digital Self-help), which is based on CBT and MI techniques and is composed of 6 modules. The intervention covers at least 30 days (5 days per module). Each module consists of an animation video, a reading assignment and an at least one writing assignment. Automated feedback is given by the program based on the results and progress from the participant. A forum and a personal diary are also available in the intervention. Based on the principles of CBT, the user is also encouraged to register their substance use or craving on a daily basis. More details about the intervention can be found in the diagram of Figure 1 and in the online document (Jellinek, 2019). Participants provided informed consent for the use of their data for research purposes when signing up for the intervention. All data was pseudonymized before the analysis, by removing all directly identifiable information such as e-mail addresses and names.

#### Definition of Success

At the start of the online intervention, the participants can choose their goal for the intervention. They can choose if they want to (gradually) stop or reduce their substance use. They can also set the target maximum daily consumption (in units) they want to reach by the end of the intervention. We use this target number of units defined by the participants combined with the prescribed use of the intervention to define success. We excluded a large number of participants who did not complete module 1 of the intervention. Based on inspection of the data, we defined intervention success as completing all 6 modules of the intervention and reaching the daily substance use goal for the last 7 days before discontinuing the intervention. We defined early dropout as completing module 1 but not going further than module 2.

#### Feature Engineering

We selected log data from the first 3 days (72 h) of intervention use of all participants. We selected this number of days after discussion with the program designers, to keep a balance between collecting as much relevant information about the usage of the intervention as possible while keeping the window for action as early as possible. Nevertheless, we explored other time windows (48 and 96 h) in a sensitivity analysis. In the first 72 h, the participants had tasks from the first module available to them, namely: watching the start video (introduction), writing pros of stopping and cons of continuing substance use (e.g., “*I will save more money if I spend less on alcohol*”), and writing agreements for themselves (e.g., “*If I reach my goal I will get myself a reward*”). Besides these tasks, other modules were available at all times such as the Forum (where the participant can interact with other participants, e.g., create, like, and reply to posts) and the diary. Daily consumption registration, logins, and forum access were also computed for the same time frame. A complete table with all the features computed and their explanation is available in Supplementary Table 1. Given the sensitive nature of the data, it is not publicly available.

#### Sensitivity Analysis

We designed experiments with a less strict definition of success, where participants that reached their target goal for at least 6 consecutive days (instead of the standard 7 of days) and finished at least 4 out of the 6 modules (instead of finishing all 6 modules) of the intervention, were also considered successful in reaching their goals. We also assessed the influence of shorter and longer time periods for data extraction (48 and 96 h). Finally, our definitions of success (finishing all modules and reaching the target consumption goal) and early dropout (reaching no further than module 2) do not include a group of participants who finish the intervention but do not achieve their target consumption goal. A total of 41% of the participants who reached module 6 of the alcohol intervention did not reach their target consumption goal and therefore, are not included in the successful group. The percentages were 33 and 39% for the cannabis and tobacco interventions, respectively. Since we defined success as a combined measure of prolonged participation and, meeting pre-set goals and given the low percentages for the cannabis and tobacco interventions (and therefore, an even smaller sample size) available in this sub-group, we did not include it in the analysis.

### Modeling

#### Machine Learning Models

Given the large number of machine learning models available in the literature, we selected a subset that has shown state-of-the-art results in recent applications. Moreover, since the learning process can be very different for each model, we aimed at including models with different learning processes to increase generalizability and the chances of new findings (Fernández-Delgado et al., 2014). Therefore, we included two models, Logistic Regression, which is often used in clinical prediction tasks, offering a linear approach and being less robust when dealing with high-dimensional data (dataset with a large number of variables), and Random Forest (Breiman, 2001), which is robust to high-dimensional datasets and can identify non-linear relationships in the data (Couronné et al., 2018). Moreover, both models offer interpretable variable importance after trained.

#### Modeling Pipeline

We used a nested k-fold cross-validation strategy to train, validate and test the models. In the outer cross-validation loop, the data was split into 10 stratified (to account for class imbalance) folds. In each cross-validation iteration, onefold was used as test set while ninefold (the training set) were used in the inner cross-validation loop. The inner cross-validation loop was used for hyper-parameter optimization, where the training data was split again into threefold, where two were used to train the model with a given set of hyper-parameters, while the one left was used to validate them. The best set of hyper-parameters was the one with the highest average Area Under the Receiver Operating Characteristic Curve (AUROC). The model trained with the best set of hyper-parameters was applied to the test set, and the evaluation measures were computed. We present a list of hyper-parameters used for model optimization in Supplementary Table 2. The participants of each substance available in the intervention were assessed separately. All code was implemented in Python 3 using the Scikit-learn library for modeling (Pedregosa et al., 2012). The code is available in the following Github repository.^1^

Class imbalance was present in all experiments and it can lead to biased results. Therefore, we applied balanced class weights during model training to address this issue (Pedregosa et al., 2012). Class weights work by multiplying the error of each sample during training. This way, classification mistakes in the minority class lead to higher loss values and a larger impact on model training. This approach has been shown to be effective even in cases of severe class imbalance (King and Zeng, 2003; Zhu et al., 2018).

#### Statistical Analysis

For each machine learning method, 10 models were optimized and tested using our nested k-fold cross-validation strategy. We report the average across all cross-validation iterations and the 95% Confidence Intervals (CI) for the following evaluation measures: AUROC, sensitivity, specificity, positive predictive value (PPV), and negative predictive value (NPV). We also present a confusion matrix with the results from all folds. The threshold of 0.5 was used for converting the probabilities to class predictions.

#### Feature Importance

For feature importance visualization we used SHAP (SHapley Additive exPlanations), which is a unified approach for explaining the predictions of any machine learning model (Lundberg et al., 2019). SHAP values are used to describe the importance a model assigns to the features for a given data point and how they influence the prediction of a certain class. SHAP allows the visualization of how high and low values of a given feature affect the prediction, offering insightful information about the models’ decision process. We opted to use SHAP instead of odds ratio from LR and Gini feature importance from RF to allow the comparison between feature importance between both models using a single model visualization tool. Moreover, Gini importance is severely biased for high cardinality features (Strobl et al., 2007), which can lead to misleading conclusions.

## Results

### Study Population

Log data from 32,398 participants enrolled from January 2016 to December 2020 in the Jellinek self-help intervention was available. Around 80% of the participants did not reach further than the first module of the intervention and were excluded from further analysis. This group of participants possibly included many individuals that only wanted to take a look at the intervention, rather than having the intention to actually follow the intervention.

The remaining 20% of the participants were divided based on the five different addiction programs available in the online intervention, namely: alcohol, cannabis, tobacco, gambling, and cocaine. Since the number of participants in the gambling and cocaine intervention was relatively low for developing machine learning models, we did not include these programs in the analysis. In Table 1 we show the total of participants included per intervention, with a total of 2,126 participants for the alcohol intervention, 466 for the cannabis intervention, and 499 for the tobacco intervention. For the alcohol intervention, from 1,085 participants that complete module 6 of the intervention, 449 did not reach their target consumption goal, and therefore, were excluded, leaving 636 participants in the successful group and 1490 in the early dropout group. For the cannabis intervention, from a total of 156 participants that finished the intervention, 52 did not reach their target consumption goal and were excluded, leaving a total of 104 participants in the successful group and 362 in the early dropout group. From the 203 participants that finished the tobacco intervention, 81 were excluded for not reaching their target goal, leaving a total of 122 participants in the successful group and 377 participants in the early dropout group.

**TABLE 1:** Distribution of participants per label definition.

Intervention	Achieve target goal	Reach module 6	Reach module 6 but not the target goal	Achieve target goal and module 6 (successful)	Dropout at module 2 (early dropout)	Total included (successful + early dropout)	Percentage include from the total participants eligible for inclusion
Alcohol	1,459	1,085	449	636	1,490	2,126	54%
Cannabis	320	156	52	104	362	466	58%
Tobacco	294	203	81	122	377	499	54%

*Early dropout (defined as finishing module 1 and reaching no further than module 2) and success (Reaching module 6 and achieving the target consumption goal). Target goal = 7 days of consumption before discontinuation. Participants eligible for inclusion are defined as anyone who reached further than module 1.*

We present in Table 2 the distribution of participants per substance used and the cumulative total of participants that reached each module. Overall, around 20% of all participants reached the second module of the intervention. This percentage differed between the substances: For alcohol, it was 26.36%, while for cannabis it was 18.50% and for tobacco, it was 9.40%.

**TABLE 2:** Distribution of participants that reach each Module and further.

Variable	All participants	Alcohol	Cannabis	Tobacco
Total start	32,398	16075	4353	9709
Removed due to missing program goal	3,573	3,188	163	222
Reach module 1	26,564 (100)	12,887 (100)	4,190 (100)	9,487 (100)
Reach module 2	5,635 (17.39)	3,905 (24.72)	807 (19.26)	923 (9.73)
Reach module 3	3,316 (10.24)	2,415 (15.28)	445 (10.62)	456 (4.81)
Reach module 4	2,395 (7.39)	1,752 (11.09)	317 (7.57)	326 (3.44)
Reach module 5	1,833 (5.66)	1,350 (8.55)	221 (5.27)	262 (2.76)
Reach module 6	1,444 (4.46)	1,085 (6.87)	156 (3.72)	203 (2.14)

*The value per module is the cumulative total, i.e., participants that reached module 6 are also counted in the previous modules. Percentages refer to the total number of participants after exclusion criteria for each substance program individually.*

Supplementary Table 3 shows the distribution of all features available per substance type and the participants split into *successful* and *early dropout* groups.

### Prediction Accuracy

We present in Table 3 the evaluation measures (mean and 95% confidence intervals) for predicting the success of participants using the online intervention for alcohol, cannabis, or tobacco. The AUROC was the highest for the alcohol and tobacco substances using the RF model (0.71–95%CI 0.69–0.73) and (0.71–95%CI 0.67–0.76), respectively. Specificity and NPV were higher than sensitivity and PPV, respectively, for all substances. The highest specificity value was for cannabis (0.78 95%CI 0.74–0.83) and for sensitivity it was for alcohol using LR (0.61 95%CI 0.57–0.65). The higher values for both specificity and NPV show that the models were better in identifying the *early dropout* participants. Prevalence of successful participation was 29, 28, and 29%, in the alcohol, cannabis and tobacco programs, respectively.

**TABLE 3:** Overall evaluation measures for the prediction of participant success for alcohol, cannabis and tobacco using Logistic Regression (LR) and Random Forest (RF).

Substance	Method	AUROC	Sensitivity	Specificity	PPV	NPV
Alcohol	LR	0.67 (0.64–0.70)	0.61 (0.57–0.65)	0.66 (0.61–0.70)	0.43 (0.40–0.47)	0.80 (0.77–0.82)
Alcohol	RF	0.71 (0.69–0.73)	0.51 (0.47–0.55)	0.77 (0.75–0.78)	0.48 (0.46–0.50)	0.79 (0.77–0.80)
Cannabis	LR	0.64 (0.58–0.70)	0.55 (0.43–0.66)	0.72 (0.67–0.77)	0.36 (0.29–0.43)	0.85 (0.82–0.88)
Cannabis	RF	0.67 (0.59–0.75)	0.47 (0.36–0.58)	0.78 (0.74–0.83)	0.38 (0.32–0.44)	0.84 (0.81–0.87)
Tobacco	LR	0.64 (0.57–0.71)	0.53 (0.44–0.63)	0.65 (0.54–0.76)	0.35 (0.27–0.44)	0.81 (0.77–0.85)
Tobacco	RF	0.71 (0.67–0.76)	0.54 (0.41–0.68)	0.76 (0.72–0.80)	0.42 (0.35–0.50)	0.84 (0.79–0.88)

*AUROC, Area under the Receiver Operating Characteristic curve; PPV, positive predictive value; NPV, negative predictive value.*

**FIGURE 2:**
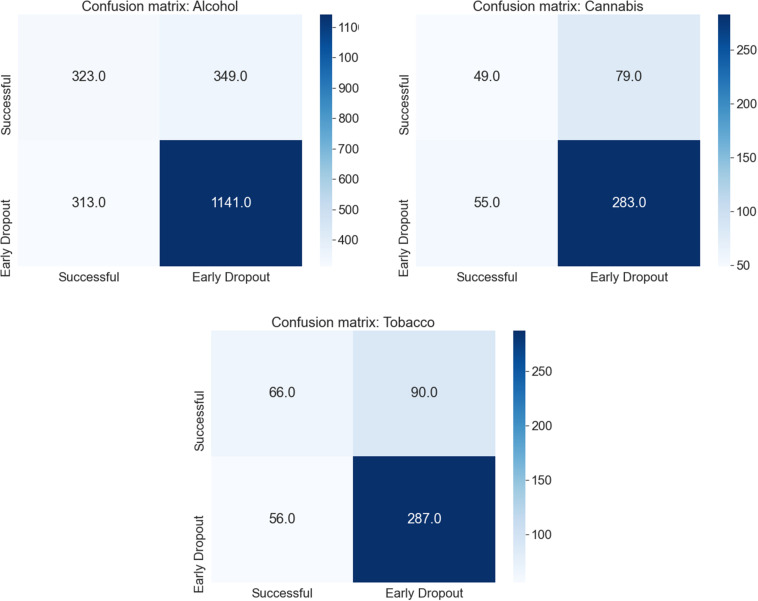
Confusion matrix for all cross-validation iterations for each substance using the Random Forest model and the standard definition of early dropout and success (reaching module 6 and 7 days of the target goal).

In Figure 2 we present the confusion matrix for each substance for the model with the highest AUROC (RF) using the results from the test sets once all cross-validation iterations were complete. For the alcohol intervention, a total of 1,141 (77%) participants with an *early dropout* outcome were correctly identified, while 323 (50%) participants with a *successful* outcome were correctly identified. The same can be observed in the cannabis intervention, where 283 (78%) participants with an *early dropout* outcome and 49 (47%) participants with a *successful* outcome were correctly identified. Finally, in the tobacco intervention, 287 (73%) participants with an *early dropout* outcome and 66 (47%) participants with a *successful* outcome were correctly classified by the RF model.

We present the results for the sensitivity analysis using other time windows for feature engineering besides 72 h (48 and 96 h) in Supplementary Table 4 and for relaxing the definition of success (achieving the target goal for 6 or 7 days before discontinuing the intervention and finishing at least 4 out of the 6 modules) in Supplementary Tables 5, 6, respectively. No differences were found when comparing the experiments with other time windows and the standard 72 h (change of around 0.01 in the average AUROC). When relaxing the definition of success (considering participants that reached 6 out of 7 days of their target goal as successful) there was a slight increase in the AUROC (around 0.01) for some interventions. Relaxing the number of target goal days to be achieved reduced the overall performance of the models. In most cases, relaxing the number of modules to be finished also led to worsening of the results (reduced prediction accuracy).

### Feature Importance

**FIGURE 3:**
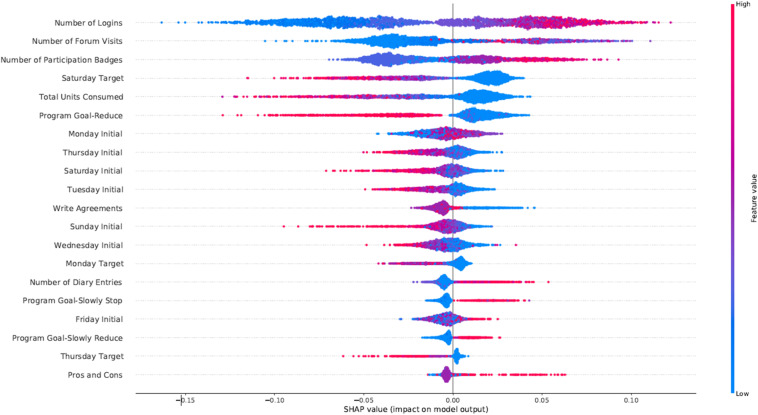
SHAP feature importance for the *alcohol* intervention using the RFC model. For visualization purposes we included only the top 20 features. In the *y*-axis, we have the features based on the first 72 h of participation in order of importance from top (most important) to bottom (less important) and in the *x*-axis their respective SHAP value which indicates their association with success (SHAP values are above zero) or early dropout (SHAP values below zero) participant outcomes.

In Figure 3 we present the feature importance (top 20 for visualization purposes) using SHAP for the RF model trained on the alcohol data. In the *y-*axis, we have the features based on the first 72 h of participation in order of importance from top (most important) to bottom (less important) and in the *x*-axis their respective SHAP value which indicates their association with *success* (SHAP values are above zero) or *early dropout* (SHAP values below zero) participant outcomes. The color legend on the right shows how large and low values of a given feature relate to the SHAP values. For example, *Number of Logins* is at the top as the most predictive feature. High values for *Number of Logins* have positive SHAP values, which indicates an association with the *success* outcome in the alcohol intervention. Most engagement-related features (*Number of Logins, Forum Visits*, and *Participation Badges*) appear at the top with high values being associated with *success*. Moreover, not drinking on the weekends (*Saturday Target*) was also associated with *success* in the alcohol intervention. The *Total Units Consumed* in the first 72 h was also considered an important predictor, with lower values associated with higher success rates. Another relevant finding is that having your target goal set to reduce (*Program Goal-Reduce*) was associated with the *early dropout* outcome for the alcohol and cannabis interventions. Finally, low initial daily consumption values (*Monday Initial, Tuesday Initial, etc.*) were associated with *success* as outcome.

**FIGURE 4:**
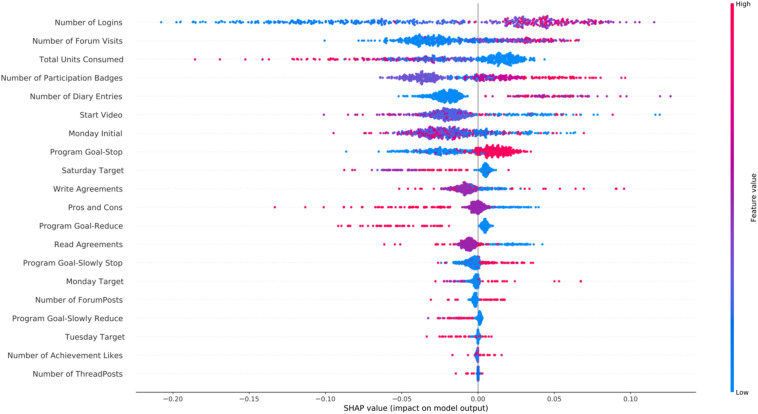
SHAP feature importance for the *cannabis* intervention using the RFC model. For visualization purposes we included only the top 20 features. In the *y*-axis, we have the features based on the first 72 h of participation in order of importance from top (most important) to bottom (less important) and in the *x*-axis their respective SHAP value which indicates their association with success (SHAP values are above zero) or early dropout (SHAP values below zero) participant outcomes.

In Figure 4 we present the SHAP feature importance plot for the cannabis intervention. The main findings for cannabis intervention were similar to the alcohol one, with the addition of the high values of the *Number of Diary Entries* being associated with participant success.

**FIGURE 5:**
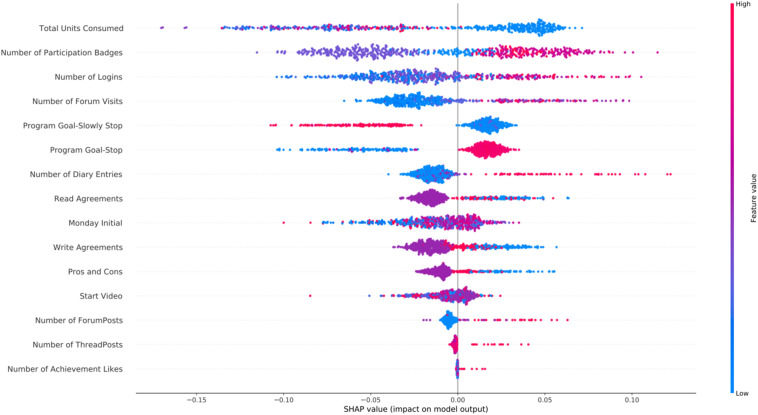
SHAP feature importance for the tobacco intervention using the RFC model. Features are shown in order of importance, from most important (top) to less important (bottom). In the *y*-axis, we have the features based on the first 72 h of participation in order of importance from top (most important) to bottom (less important) and in the *x*-axis their respective SHAP value which indicates their association with success (SHAP values are above zero) or early dropout (SHAP values below zero) participant outcomes.

Figure 5 shows the SHAP feature importance plot for the tobacco intervention. Since many features were highly correlated with each other they were removed from the analysis. Moreover, consumption target variables were not included since for the tobacco intervention only (slowly) quitting is an option (instead of the other options of reducing or slowly reducing available in the other interventions), therefore all target goals are set to zero. A total of 15 features were included in the models and in Figure 5. The *Total Units Consumed* was the most important variable for the tobacco intervention, with low values being associated with *success*. Having the target goal set to stop instead of slowly stop was also an important predictor. Finally, as also observed in the other interventions, engagement variables such as *Number of Logins, Forum Visits* and *Number of Participation Badges* were highly predictive with high values associated with success.

## Discussion

We have shown that machine learning models can accurately identify participants that will be successful in reaching their goals in the online self-help intervention for alcohol, cannabis, and tobacco. The best AUROC values were 0.71, 0.67, and 0.71 for the alcohol, cannabis, and tobacco interventions, respectively, which shows moderate predictive value. Despite all models having similar performance, the AUC was the lowest for the cannabis intervention. This is likely due to the small number of samples available for training since this was also the intervention with the least participants. Moreover, the high negative predictive values reported suggests that our models were better at identifying participants at risk of early dropout, while the high specificity shows that the early dropout predictions were often correct. Such findings could be used in practice, to offer extra support for this risk group. We have identified that engagement with the intervention, alongside with having the target goal to quit immediately instead of gradually quitting or moderating substance use, and not drinking on the weekends were all important predictors of participant *success*. Thus, our findings have important implications for implementation trials geared at increasing adherence and success in ATOD self-help programs.

The prediction of participant adherence and success to addiction treatments (including CBT) has been previously explored in the literature (Acion et al., 2017; Symons et al., 2019; van Emmerik-van Oortmerssen et al., 2020) since it can lead to new insights and subsequently to improvements to the service. The prediction of dropout and outcome of a CBT treatment for Attention Deficit Hyperactivity Disorder and Substance Use Disorders (SUDs) was investigated by van Emmerik-van Oortmerssen et al. (2020). They found a significant association between participant demographics variables and drop-out from CBT. Despite their positive findings, the number of participants included was relatively small (119) and only linear models were explored. Acion et al. (2017) investigated the use of machine learning for predicting SUD treatment success. They included a large population (99,013 participants) and reported AUROCs up to 0.82. Nevertheless, their work was limited to in-hospital treatment (no CBT) and defined success as only reaching the end of treatment (i.e., an adherence goal). In the study from Symons et al. (2019) machine learning models were used to predict treatment outcomes of a CBT treatment for alcohol dependence. Demographics and psychometric data from 780 participants were included in the models, and they reported very low accuracy results, with AUROC values around 0.50 (close to random). The prediction of outcome of a CBT for tobacco smoking was explored in a study by Coughlin et al. (2020), where demographics and impulsivity measures were used to train a decision tree model and an accuracy of 74% was reported in the validation set. Our study builds upon and extends previous studies by including a large population of 32,398 participants from a self-help digital intervention for SUD, by using a nested cross-validation strategy to reduce the risk of biased results, including multiple variables available in the log data from the intervention (instead of commonly used demographics), by using non-linear models in the analysis pipeline, and by reporting the importance of feature values in the models’ decision process. Future experimental work should clarify whether found predictors are true risk factors in that they independently contribute to success, or are only risk markers.

### Clinical Interpretation

Our results suggest that users of the Jellinek online intervention that are more engaged (and probably more motivated) with the intervention in the first 3 days of participation tend to reach their goals more often. Having the goal of immediately quitting rather than gradually quitting or moderating use, and the goal to quit using substances on the weekends also correlates with success. These findings correspond with previous studies that investigated differences between gradually vs. abruptly quitting (Cheong et al., 2007; Hughes et al., 2010; Lindson-Hawley et al., 2016), while they tap into an old and wider discussion on whether either moderation or cessation are valid and feasible substance use treatment goals (Owen and Marlatt, 2001; Cheong et al., 2007; Hughes et al., 2010; Luquiens et al., 2011; Lindson-Hawley et al., 2016; Haug et al., 2017). Moreover, in the tobacco intervention, the *Total Units Consumed* was the most important predictor, while in the alcohol and cannabis interventions, it was the *Number of Logins.* The reasoning behind this difference is not entirely clear, but a possibility is that it is related to the possible goals of each intervention. For tobacco, only completely quitting can lead to a successful outcome. Therefore, the association between tobacco use quantities and success is much stronger, as success can only be achieved when tobacco use goes to 0 at some point. For alcohol/cannabis, this association is slightly less strong as moderate use of these substances (target goal set to reduce) can still lead to a successful outcome and goal achievement. Finally, quitting is more difficult for heavy smokers than for people who smoke less, making it a strong predictor of success (Vangeli et al., 2011).

Therefore, surveying patients on their motivation before the start of the intervention, extending self-therapy by incentivizing daily use of the program, for instance, by interactive gaming mechanisms (“gamification”) and/or availability of novel assignments each day, and encouraging therapy continuation by positive feedback on their progress, may have a positive effect for participants. Explaining how choices between quitting and controlled use as a treatment goal, and in case of controlled use, how substance use during weekends may affect their goal attainment might assist the participant in making better-informed decisions, ultimately leading to improved outcomes. Such amendments to the intervention and how these might affect participant success will be the topic of future studies. Finally, regarding the methods, one could consider different optimization strategies or probability cut-offs to prioritize the identification of either *early dropouts* or *successful* participants. In our case, we aimed at prioritizing the correct identification of participants at risk of early dropout (NPV), which was higher for the standard 0.5 cut-off, while other approaches such as the Youden Index (Youden, 1950) led to more balanced sensitivity and specificity values.

### Strengths and Limitations

A strong point of this study is the large sample size, which includes all participants that used the intervention since 2016 and indicated their data may be used for research purposes, making the data to a large extent representative of the full population that participates in the Jellinek ATOD online intervention. Our approach included multiple validation steps to reduce the risk of overfitting and biased results. Furthermore, we increased model transparency by using SHAP for model interpretation, which makes our results clearer and more actionable. A limitation of this study is the lack of demographic variables since these have previously been shown to be strong predictors of participant success (van Emmerik-van Oortmerssen et al., 2020). However, given the fact that demographics are not changeable, they cannot result in research aimed at improving treatment success, limiting their practical value. Due to privacy concerns, variables such as age, sex, and highest degree achieved are not mandatory for the participants to fill in during registration and are, therefore, highly missing in our dataset (more than 80%). Another limitation is the large number of participants that do not even finish the first module from the intervention and were excluded from this study. The large number of early dropouts is quite common in unguided self-help interventions, and finding predictors for it is often difficult (Eysenbach, 2005; Beatty and Binnion, 2016). Since our main goal was to predict success based on log data, a minimum number of days of use was necessary to make such data available. Nevertheless, the excluded participants represent a significant part of the population, which can impact the generalizability of our results. Future studies are necessary to explore the reasons behind the large number of participants that seem to barely use the intervention before dropping out. The group of participants that finished the intervention but did not reach their target goal was excluded from our analysis since they did not match our definition of success. This is a limitation of our study since despite being small, this is an important group that seems to be motivated enough to go all the way through the intervention, while not fully benefiting from it. Moreover, provided more data for such group is available in the future, a model capable of differentiating between participants that, despite finishing the intervention, will reach their target consumption goals or not, could be of great assistance. Finally, in all experiments, accuracy was relatively limited, with AUROCs around 0.70. The evaluation measures were higher for the substances with more participants, which suggests that our results could improve if more data would be available.

## Conclusion

Log data analysis with machine learning yielded positive results for the prediction of participant success in the digital self-help Jellinek intervention, with the models being especially accurate in predicting patients at risk of early discontinuation. We also identified multiple relevant predictors of outcomes and how participants’ choices regarding goals may affect goal achievement. Whether this information can lead to improvements to the intervention will be the subject of future studies.

## Data Availability Statement

The data analyzed in this study is subject to the following licenses/restrictions: Given the sensitive nature of the data, and to preserve the identity of the participants the data cannot be made publicly available. Requests to access these datasets should be directed to MB.

## Ethics Statement

Ethical review and approval was not required for the study on human participants in accordance with the local legislation and institutional requirements. The patients/participants provided their written informed consent to participate in this study.

## Author Contributions

LR: lead author, study design, analysis and interpretation of results, and critical revision of the manuscript. MB: data extraction, study design, analysis and interpretation of results, and decision making and critical revision of manuscript. GW and SP: study design, analysis and interpretation of results, and decision making and critical revision of manuscript. TB: result interpretation, analysis and interpretation of results, and decision making and critical revision of manuscript. AG: study design, analysis and interpretation of results, and decision making and critical revision of the manuscript. All authors contributed to the article and approved the submitted version.

## Conflict of Interest

The authors declare that the research was conducted in the absence of any commercial or financial relationships that could be construed as a potential conflict of interest.

## Publisher’s Note

All claims expressed in this article are solely those of the authors and do not necessarily represent those of their affiliated organizations, or those of the publisher, the editors and the reviewers. Any product that may be evaluated in this article, or claim that may be made by its manufacturer, is not guaranteed or endorsed by the publisher.

## Footnotes

1
https://github.com/L-Ramos/ML_ehealth
