# Supplementary material for: Predicting Success of a Digital Self-Help Intervention for Alcohol and Substance Use With Machine Learning
Source: Front Psychol. 2021 Sep 3;12:734633. doi: 10.3389/fpsyg.2021.734633 (PMC8451420; doi:10.3389/fpsyg.2021.734633)
Supplement: Supplementary file 1 [file Table_1.DOCX]

Supplementary Material

Supplementary Table 1 - Description of all variables included in the models and their data type.

| Variable | Description | Type |
| --- | --- | --- |
| Start Video* | Number of time the start video assignment was done. Provides an introduction to the intervention. | Continuous |
| Read Agreements* | Number of times the reading assignment was done. Provides an introduction to agreements the user should do with himself. | Continuous |
| Pros and Cons* | Number of times the pros and cons assignment was done. The user is supposed to write the pros of stopping and the cons of continuing substance use. | Continuous |
| Write Agreements* | Number of times the write agreement assignment was done. The user should write some agreements for changes he is willing to make. | Continuous |
| *Day-of-the-week* Initial | The user fills in at the start of the intervention how many units of the substance he/she consumes each day of the week. | Continuous |
| *Day-of-the-week* Target | The user fills in at the start of the intervention how many units of the substance he/she would like to consume per day at the end of the intervention. | Continuous |
| Number of Forum Visits | Number of times the user visited the forum section. | Continuous |
| Number of Logins | Number of times the user logged in the intervention. | Continuous |
| Number of Forum Posts | Number of times the user created a post in forum section. | Continuous |
| Number of Thread Posts | Number of times the user created a thread in the forum section | Continuous |
| Number of Participation Badges | Number of badges the user earned by doing the assignments. | Continuous |
| Number of Achievement Likes | Number of achievements the user received (and read) by reaching certain milestones. | Continuous |
| Total Units Consumed | Total number of units the user reported to have consumed in the first (3) days of the intervention. | Continuous |
| Number of Diary Entries | Total number of diary entries by the user. | Continuous |
| Program Goal: Reduce** | Overall target goal set by the user at the start of the intervention. Reduce means reducing consumption. | Categorical |
| Program Goal: Slowly Reduce** | Overall target goal set by the user at the start of the intervention. Slowly reduce means reducing consumption but at a slower pace during the intervention. | Categorical |
| Program Goal: Stop | Overall target goal set by the user at the start of the intervention. Stop means stopping consumption at the end of the intervention. | Categorical |
| Program Goal: Slowly Stop | Overall target goal set by the user at the start of the intervention. Slowly stop means stopping consumption but at a slower pace during the intervention. | Categorical |
| *Users are supposed to do the assignment at least once, but can revisit them multiple times.  **Options not available for the smoking intervention | | |

Supplementary Table 2 - Hyper-parameters used for optimizing the Machine Learning models using grid-search.

| **Classifier** | **Parameter Name** | **Parameter Value** |
| --- | --- | --- |
| **RFC** | Number of Trees | [100,200,400,600,800,1000,1200,1400] |
|  | Max features for split | auto, sqrt and log2 |
|  | Max depth of trees | [10,20,30,40, 50, 60, 70, 80, 90, 100, None] |
|  | Quality of split | Gini or Entropy |
|  | Minimum number of samples required to split an internal node | [2,4,6,8] |
|  | Minimum number of samples required to be at a leaf node | [2,4,6,8,10] |
| **LR** | Regularization | [0.001, 0.01**,** 0.1, 1, 10, 100] |
|  | Optimization algorithm | [newton-cg, lbfgs, liblinear, sag, saga] |

Table 3 - Variable distribution per substance type split in successful and early dropout groups. Median and 25-75% interquartile ranges are reported for the continuous variables and the total number followed by the percentage for categorical ones.

| **Variable** | **Alcohol early dropout N = 1490** | **Alcohol Successful N = 636** | **Cannabis early dropout  N = 362** | **Cannabis Successful N = 104** | **Tobacco early dropout  N = 377** | **Tobacco Successful N = 122** |
| --- | --- | --- | --- | --- | --- | --- |
| Start Video | 2 (2 - 2) | 2 (2 - 3) | 2 (2 - 2) | 2 (2 - 3) | 2 (2 - 2) | 2 (2 - 2) |
| Read Agreements | 1 (1 - 1) | 1 (1 - 1) | 1 (1 - 1) | 1 (1 - 1) | 1 (1 - 1) | 1 (1 - 1) |
| Pros and Cons | 1 (1 - 1) | 1 (1 - 1) | 1 (1 - 1) | 1 (1 - 1) | 1 (1 - 1) | 1 (1 - 1) |
| Write Agreements | 1 (1 - 1) | 1 (1 - 1) | 1 (1 - 1) | 1 (1 - 1) | 1 (1 - 1) | 1 (0 - 1) |
| Monday Initial | 4 (2 - 6) | 4 (2 - 6) | 3 (2 - 5) | 3 (1 - 5) | 15 (7 - 20) | 15 (6 - 20) |
| Tuesday Initial | 3 (0 - 5) | 3 (0 - 5) | 2 (1 - 4) | 3 (1 - 4) | 15 (8 - 20) | 13 (8 - 20) |
| Wednesday Initial | 3 (0 - 5) | 3 (0 - 5) | 2 (1 - 4) | 2 (1 - 4) | 15 (8 - 20) | 12 (6 - 20) |
| Thursday Initial | 4 (0 - 5) | 3 (0 - 5) | 2 (1 - 4) | 2 (1 - 4) | 15 (8 - 20) | 14 (6 - 20) |
| Friday Initial | 4 (2 - 6) | 3 (0 - 6) | 2 (1 - 4) | 2 (1 - 4) | 15 (8 - 20) | 12 (5 - 20) |
| Saturday Initial | 5 (4 - 8) | 5 (3 - 7) | 3 (2 - 5) | 3 (2 - 4) | 15 (8 - 20) | 12 (6 - 20) |
| Sunday Initial | 6 (4 - 8) | 5 (3 - 8) | 3 (2 - 5) | 3 (2 - 5) | 15 (8 - 22) | 15 (7 - 20) |
| Monday Target | 0 (0 - 2) | 0 (0 - 0) | 0 (0 - 0) | 0 (0 - 0) | NA | NA |
| Tuesday Target | 0 (0 - 0) | 0 (0 - 0) | 0 (0 - 0) | 0 (0 - 0) | NA | NA |
| Wednesday Target | 0 (0 - 0) | 0 (0 - 0) | 0 (0 - 0) | 0 (0 - 0) | NA | NA |
| Thursday Target | 0 (0 - 0) | 0 (0 - 0) | 0 (0 - 0) | 0 (0 - 0) | NA | NA |
| Friday Target | 0 (0 - 0) | 0 (0 - 0) | 0 (0 - 0) | 0 (0 - 0) | NA | NA |
| Saturday Target | 0 (0 - 2) | 0 (0 - 2) | 0 (0 - 0) | 0 (0 - 0) | NA | NA |
| Sunday Target | 0 (0 - 3) | 0 (0 - 2) | 0 (0 - 0) | 0 (0 - 0) | NA | NA |
| Number of Forum Visits | 0 (0 - 3) | 2 (0 - 6) | 0 (0 - 2) | 1 (0 - 3) | 0 (0 - 2) | 1 (0 - 3) |
| Number of Logins | 4 (3 - 6) | 6 (4 - 8) | 3 (2 - 5) | 4 (3 - 6) | 4 (2 - 5) | 4 (2 - 7) |
| Number of Forum Posts | 0 (0 - 0) | 0 (0 - 0) | 0 (0 - 0) | 0 (0 - 0) | 0 (0 - 0) | 0 (0 - 0) |
| Number of Thread Posts | 0 (0 - 0) | 0 (0 - 0) | 0 (0 - 0) | 0 (0 - 0) | 0 (0 - 0) | 0 (0 - 0) |
| Number of Participation Badges | 1 (1 - 2) | 2 (1 - 3) | 1 (1 - 2) | 2 (1 - 2) | 1 (1 - 2) | 2 (1 - 2) |
| Number of Achievement Likes | 0 (0 - 0) | 0 (0 - 0) | 0 (0 - 0) | 0 (0 - 0) | 0 (0 - 0) | 0 (0 - 0) |
| Total Units Consumed | 0 (0 - 6) | 0 (0 - 4) | 1 (0 - 4) | 0 (0 - 3) | 2 (0 - 19) | 0 (0 - 10) |
| Number of Diary Entries | 0 (0 - 0) | 0 (0 - 1) | 0 (0 - 0) | 0 (0 - 1) | 0 (0 - 0) | 0 (0 - 1) |
| Program Goal: Reduce* | 506 (33.96) | 143 (22.48) | 43 (11.88) | 5 (4.81) | NA | NA |
| Program Goal: Slowly Reduce* | 295 (19.80) | 109 (17.14) | 41 (11.33) | 8 (7.69) | NA | NA |
| Program Goal: Slowly Stop* | 166 (11.14) | 108 (16.98) | 82 (22.65) | 19 (18.27) | 130 (34.48) | 21 (17.21) |
| Program Goal: Stop* | 523 (35.10) | 276 (43.40) | 196 (54.14) | 72 (69.23) | 247 (65.52) | 101 (82.79) |
| *Categorical variables: total and percentages are reported. | | | | | | |

Supplementary Table 4 - Overall evaluation measures for the sensitivity analysis (using 48 and 96 hours as time window for feature engineering) for predicting participant success in the alcohol, cannabis and smoking interventions using Random Forest (RF). PPV: positive predictive value; NPV: negative predictive value.

| **Program** | **Time window** | **AUC 95% CI** | **Sensitivity** | **Specificity** | **PPV** | **NPV** |
| --- | --- | --- | --- | --- | --- | --- |
| Alcohol | 48 | 0.69  (0.66 - 0.71) | 0.51  (0.47 - 0.54) | 0.76  (0.74 - 0.78) | 0.47  (0.45 - 0.50) | 0.78  (0.77 - 0.80) |
| Cannabis | 48 | 0.65  (0.55 - 0.74) | 0.42  (0.31 - 0.54) | 0.78  (0.72 - 0.85) | 0.37  (0.29 - 0.44) | 0.83  (0.79 - 0.86) |
| Tobacco | 48 | 0.66  (0.61 - 0.72) | 0.43  (0.35 - 0.51) | 0.73  (0.68 - 0.77) | 0.34  (0.28 - 0.39) | 0.80  (0.77 - 0.82) |
| Alcohol | 96 | 0.72  (0.70 - 0.75) | 0.54  (0.50 - 0.58) | 0.79  (0.76 - 0.81) | 0.52  (0.49 - 0.55) | 0.80  (0.78 - 0.81) |
| Cannabis | 96 | 0.68  (0.60 - 0.76) | 0.46  (0.33 - 0.60) | 0.77  (0.71 - 0.84) | 0.37  (0.28 - 0.46) | 0.84  (0.80 - 0.87) |
| Tobacco | 96 | 0.71  (0.66 - 0.76) | 0.57  (0.47 - 0.67) | 0.75  (0.70 - 0.79) | 0.42  (0.36 - 0.48) | 0.84  (0.81 - 0.88) |

Supplementary Table 5 – Overall evaluation measures for the sensitivity analysis (6 out of 7 days of the target goal achieved with last phased reached by the participants varying from 4 to 6) for predicting participant success in the alcohol, cannabis and smoking interventions using Random Forest (RF). PPV: positive predictive value; NPV: negative predictive value.

| **Program** | **Min Phase** | **AUC 95% CI** | **Sensitivity** | **Specificity** | **PPV** | **NPV** |
| --- | --- | --- | --- | --- | --- | --- |
| Alcohol | 4/6 | 0.66  (0.64 - 0.68) | 0.44  (0.41 - 0.46) | 0.74  (0.72 - 0.76) | 0.40  (0.37 - 0.42) | 0.78  (0.77 - 0.79) |
| Alcohol | 5/6 | 0.68  (0.66 - 0.70) | 0.46  (0.42 - 0.50) | 0.76  (0.74 - 0.78) | 0.41  (0.38 - 0.44) | 0.80  (0.79 - 0.81) |
| Alcohol | 6/6 | 0.69  (0.67 - 0.71) | 0.43  (0.38 - 0.49) | 0.80  (0.77 - 0.82) | 0.41  (0.38 - 0.44) | 0.81  (0.80 - 0.82) |
| Cannabis | 4/6 | 0.62  (0.56 - 0.67) | 0.39  (0.29 - 0.48) | 0.72  (0.66 - 0.78) | 0.32  (0.24 - 0.39) | 0.78  (0.75 - 0.81) |
| Cannabis | 5/6 | 0.65  (0.58 - 0.72) | 0.44  (0.32 - 0.56) | 0.78  (0.74 - 0.83) | 0.36  (0.30 - 0.42) | 0.83  (0.80 - 0.86) |
| Cannabis | 6/6 | 0.67  (0.64 - 0.70) | 0.37  (0.26 - 0.47) | 0.81  (0.77 - 0.85) | 0.32  (0.22 - 0.41) | 0.84  (0.81 - 0.86) |
| Smoking | 4/6 | 0.66  (0.60 - 0.71) | 0.48  (0.34 - 0.63) | 0.74  (0.70 - 0.77) | 0.37  (0.31 - 0.44) | 0.81  (0.77 - 0.86) |
| Smoking | 5/6 | 0.66  (0.60 - 0.72) | 0.50  (0.41 - 0.60) | 0.73  (0.68 - 0.79) | 0.38  (0.33 - 0.43) | 0.83  (0.80 - 0.85) |
| Smoking | 6/6 | 0.69  (0.64 - 0.73) | 0.52  (0.47 - 0.58) | 0.73  (0.68 - 0.77) | 0.35  (0.31 - 0.39) | 0.85  (0.83 - 0.86) |

Supplementary Table 6 – Overall evaluation measures for the sensitivity analysis (7 out of 7 days of the target goal achieved with last phased reached by the participants varying from 4 to 6) for predicting participant success in the alcohol, cannabis and smoking interventions using Random Forest (RF). PPV: positive predictive value; NPV: negative predictive value.

| **Program** | **Min Phase** | **AUC 95% CI** | **Sensitivity** | **Specificity** | **PPV** | **NPV** |
| --- | --- | --- | --- | --- | --- | --- |
| Alcohol | 4/6 | 0.69  (0.67 - 0.72) | 0.56  (0.53 - 0.59) | 0.71  (0.69 - 0.74) | 0.54  (0.51 - 0.57) | 0.73  (0.71 - 0.74) |
| Alcohol | 5/6 | 0.70  (0.67 - 0.73) | 0.52  (0.48 - 0.56) | 0.75  (0.73 - 0.77) | 0.51  (0.48 - 0.54) | 0.76  (0.74 - 0.77) |
| Alcohol | 6/6 | 0.71  (0.69 - 0.73) | 0.51  (0.47 - 0.55) | 0.77  (0.75 - 0.78) | 0.48  (0.46 - 0.50) | 0.79  (0.77 - 0.80) |
| Cannabis | 4/6 | 0.68  (0.59 - 0.77) | 0.50  (0.33 - 0.66) | 0.68  (0.62 - 0.74) | 0.41  (0.32 - 0.49) | 0.75  (0.68 - 0.82) |
| Cannabis | 5/6 | 0.67  (0.63 - 0.71) | 0.47  (0.39 - 0.55) | 0.74  (0.67 - 0.81) | 0.40  (0.34 - 0.46) | 0.80  (0.78 - 0.82) |
| Cannabis | 6/6 | 0.67  (0.59 - 0.75) | 0.47  (0.36 - 0.58) | 0.78  (0.74 - 0.83) | 0.38  (0.32 - 0.44) | 0.84  (0.81 - 0.87) |
| Tobacco | 4/6 | 0.69  (0.63 - 0.75) | 0.54  (0.44 - 0.64) | 0.76  (0.70 - 0.81) | 0.51  (0.43 - 0.58) | 0.79  (0.75 - 0.82) |
| Tobacco | 5/6 | 0.72  (0.65 - 0.78) | 0.53  (0.44 - 0.61) | 0.76  (0.69 - 0.82) | 0.47  (0.39 - 0.54) | 0.80  (0.78 - 0.83) |
| Tobacco | 6/6 | 0.71  (0.67 - 0.76) | 0.54  (0.41 - 0.68) | 0.76  (0.72 - 0.80) | 0.42  (0.35 - 0.50) | 0.84  (0.79 - 0.88) |
